# Metal-Oxide FET Biosensor for Point-of-Care Testing: Overview and Perspective

**DOI:** 10.3390/molecules27227952

**Published:** 2022-11-17

**Authors:** Mohamed Taha Amen, Thuy Thi Thanh Pham, Edward Cheah, Duy Phu Tran, Benjamin Thierry

**Affiliations:** Future Industries Institute, University of South Australia, Mawson Lakes Campus, Adelaide, SA 5000, Australia

**Keywords:** point-of-care testing, field effect transistor sensor, semiconductor materials, metal-oxide, regulatory pathway

## Abstract

Metal-oxide semiconducting materials are promising for building high-performance field-effect transistor (FET) based biochemical sensors. The existence of well-established top-down scalable manufacturing processes enables the reliable production of cost-effective yet high-performance sensors, two key considerations toward the translation of such devices in real-life applications. Metal-oxide semiconductor FET biochemical sensors are especially well-suited to the development of Point-of-Care testing (PoCT) devices, as illustrated by the rapidly growing body of reports in the field. Yet, metal-oxide semiconductor FET sensors remain confined to date, mainly in academia. Toward accelerating the real-life translation of this exciting technology, we review the current literature and discuss the critical features underpinning the successful development of metal-oxide semiconductor FET-based PoCT devices that meet the stringent performance, manufacturing, and regulatory requirements of PoCT.

## 1. Introduction

Point-of-care testing (PoCT) describes a diagnostic or prognostic procedure conducted close to or at the site of a patient. It aims to deliver reliable and objective results and, consequently, to improve patient management and/or care [1,2]. PoCT technology should ideally be fast-preferably with a time-to-result of less than an hour, inexpensive, portable, and instrument-free. Importantly, it should be preferably easily performed by primary healthcare providers or even patients with no specific training [3,4]. As illustrated by the COVID-19 pandemic, the availability of PoCT diagnostic tests is a pressing need to address existing and emerging healthcare problems. This need is particularly critical for low-resource countries that are often characterized by inadequate or lacking laboratory facilities and limited availability of trained staff [5,6]. In high-resource countries, PoC technologies are progressing and are already important for the detection of conditions including strokes [7], heart failure [8], brain injury [9], preeclampsia [10,11], and sepsis [12]. In addition, due to its ability to provide immediate results in non-laboratory settings, PoCT can also be deployed in disaster and/or remote areas [2].

While many PoCT approaches have relied to date on well-established technologies such as lateral flow assays, there is an unquestionable need for more performance PoCT. For example, quantitative insight into the concentration of one or more biomarkers is required in many cases to establish a reliable diagnostic. This is challenging, especially for analytes present at low concentrations in the samples. Recent advances in nanotechnology and molecular sciences are broadly anticipated to provide implementable solutions to this challenge in the form of nanoscale biosensors, which present significant advantages for the PoCT platform [13,14].

**Figure 1:**
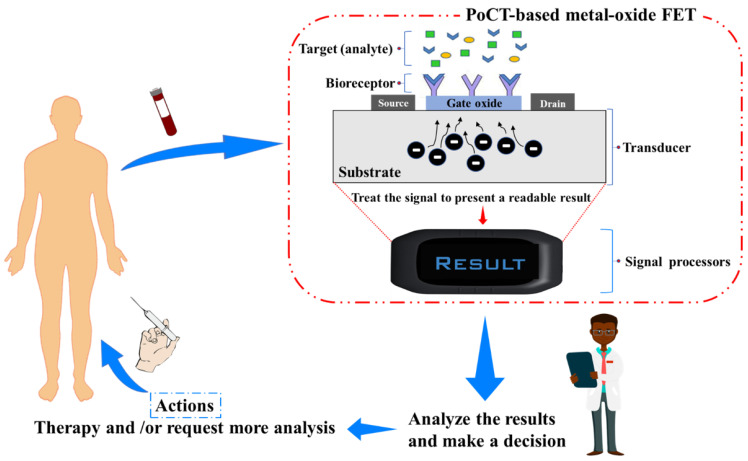
Illustration of a typical PoCT-based metal-oxide field-effect transistor biosensor and its operation process.

Conceptually, a biosensor is an analytical device that converts the input variable into a measurable signal using biological recognition elements that integrate within or intimately with a physicochemical transducer element [15]. Typically, a biosensor consists of three components: the biological recognition element (bioreceptors), the transducer, and microelectronics (signal processors), as illustrated in Figure 1. Among the different types of transducers, nanoscale field-effect transistors (FETs) are a prime candidate for PoCT owing to their excellent analytical performance and ultralow limit of detection (LOD), fast detection time, direct electrical signal transduction, manufacturing scalability, and integrability within PoC sampling platform [16,17,18,19]. Materials that have been explored for nano FETs-based biochemical sensors include silicon [20,21,22], metal-oxides [14,23,24,25,26], III-V materials [27,28,29], polymers [30,31,32], and graphene and carbon nanotubes [33,34,35,36]. Among these materials, metal-oxide semiconductor materials possess a combined set of advantages in the context of PoCT. These include the fact that metal-oxide semiconductor-based nano-FET sensors offer high biosensing performance, with a typical detection limit in the fg/mL range [14,24,25,37]. In addition, due to their wide bandgap nature, their electrical properties are only moderately influenced by minor changes in the sensing environment (e.g., temperature, light). The Fabrication of metal oxide nanoscale FET and their packaging are relatively straightforward and scalable and do not require advanced fabrication facilities, making them compatible with up-scaled production [26,38,39,40].

We review the current literature and discuss the key aspects relevant to the development of metal-oxide-based FET for PoCT devices. We first present an overview of the most common wide-band gap metal-oxide semiconductors and discusses important selection criteria for creating successful FET-based PoCT devices. We then describe the main fabrication approaches and challenges associated with integrating metal-oxide-based-FETs. Next, the key yet often neglected issue of sample pre-processing is discussed. Finally, we briefly introduce the regulatory landscape of PoCT technology.

## 2. Nano-FET Biosensor-Based Metal-Oxide Semiconductor Materials

### 2.1. Overview of Metal-Oxide Semiconductor Materials

Several metal-oxide semiconductor materials have been used as active elements in FETs [41,42]. The most common ones, along with their respective electrical properties and examples of biosensing applications, are summarized in Table 1. Several criteria should be considered when selecting a metal-oxide semiconductor for chemical/biosensor FET-based PoCT. First, the specific applications should be considered. For instance, target concentration levels and diagnostically relevant cut-offs, sample nature (e.g., blood, saliva, urine, sweat), singleplex or multiplexed measurement, and intended implementation settings (e.g., remote/low resource area, emergency department). These considerations influence the selection of the most suitable materials and designs. Second, metal-oxide semiconductor materials’ commercial availability and their electrical/mechanical stability in the measurement environment. For example, in the case of gas/organic molecules sensing for a breath analyzer, where a high temperature is usually required, both the semiconductor and the metallization material must be able to withstand that temperature. On the other hand, the material must be sufficiently stable in the intended solution for wet-chemical and biological sensing. In addition, the semiconductor material’s sensitivity to light and temperature should also be considered. For example, materials sensitive to visible light require stringent packaging to be isolated and/ or to be operated in the dark. Generally, high bandgap semiconductor materials are less sensitive to light and temperature [26,43]. Finally, and importantly, compatibility with upscale manufacturing is also critical to real-life translation. Among possible metal-oxide semiconductor materials, indium oxide, indium tin oxide, zinc oxide, and tin oxide are the most widely used metal-oxides for FET-based biosensors because of their superior electrical characteristics, chemical stability, and easiness of fabrication.

### 2.2. Operation of Metal-Oxide FET-Based Biosensor

As illustrated in Table 1, several types of analytes/biomarkers (e.g., antigen, nucleic acid, virus and virus protein/capsid, bacteria) have been successfully implemented in metal-oxide FET sensors. A FET-based biosensor typically relies on the integration of an ISFET and bioreceptors with suitable binding affinity and specificity to the target analyte. The nature of the target analyte influences the design of the overall assay and detection mechanisms. As with other FET sensors, different classes of bioreceptors can be used (see also Section 3.3), the most common being antibodies and antibody-fragments, enzymes, nucleic acid-based probes, and aptamers. Bioreceptors are typically immobilized on the semiconductor material (the sensing channel) and display biding sites to capture the target analyte(s). The surface potential of the FET sensors and, therefore, the channel conductance changes when these bioreceptors bind with the targets. The channel conductance variation resulting from the presence of the target can be correlated to a sensitivity index by measuring the changes in the drain current. The presence of the target on the sensing channel is typically detected either directly (label-free operation) or via a secondary amplification step. In addition, competitive and displacement affinity assays can also be used, with or without amplification.

Label-free assays rely on the intrinsic charges present on the target at the measurement condition [15,74] or conformational changes induced by its binding and are conceptually easier to design and implement than two-step sandwich assays. However, the issue of the sensor non-specific fouling by biomolecules present in the test matrix typically imposes a limit on the analytical performance. In addition, it is worth mentioning that the Debye length, which governs the extent to which the analytes’ charges affect the FET channel electrical surface characteristics, should also be considered, as it severely limits the direct detection of the analytes in physiological solutions. Various approaches have been proposed to overcome the Debye length effect. For example, an oligonucleotide stem-loop bioreceptor was successfully used for adaptive target recognition in ultrathin In_2_O_3_ FETs [55]. Another approach modulated the Debye screening length without affecting the FET’s channel surface by fabricating a Meta-Nano-Channel Bio-FET to tune the double-layer shielding electrostatically [75]. In addition, the integration of an extended gate with an optimized FET design could address the Debye effect [76,77].

**Figure 2:**
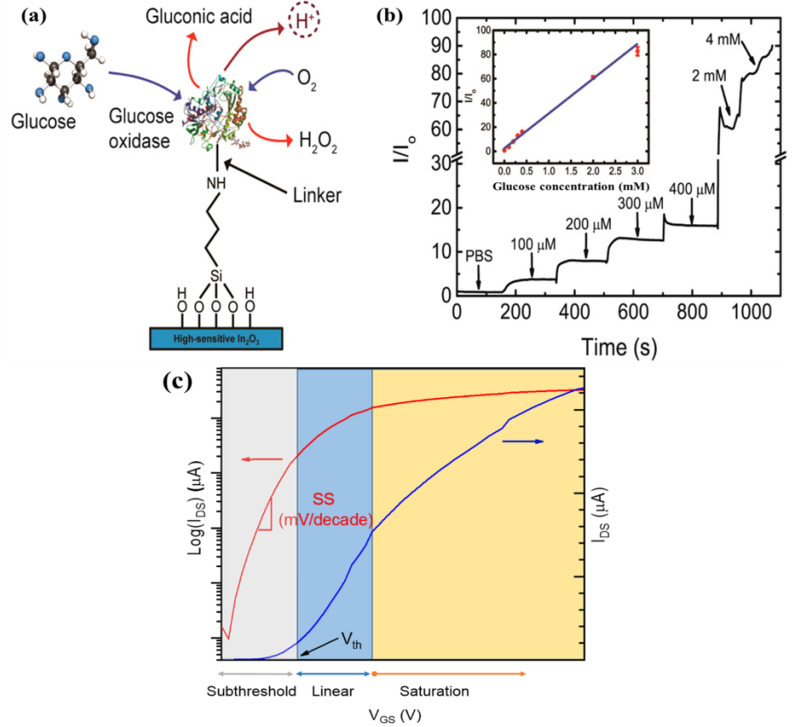
(**a**) Oxidation process of D-glucose by glucose oxidase enzyme produces protons that can modify the surface potential of an indium oxide FET; (**b**) Indium oxide FET electrical response to D-glucose concentrations in human diabetic tears (lower range) and blood (upper range), lower range, and upper range, respectively. Adapted from [78]. Copyright © 2015, American Chemical Society; (**c**) Different regimes of FET operation.

Signal amplification is typically achieved via an enzymatic reaction, either directly if the bioreceptor is an enzyme or indirectly via a secondary probe conjugated with an enzyme. Direct enzymatic assays are easier to implement than two-step sandwich assays; however, only a limited number of analyte/bioreceptor pairings are available. Figure 2a,b illustrates a direct enzymatic assay for ultra-thin indium oxide FET biosensors functionalized with glucose oxidase as a selective bioreceptor for D-glucose, yielding ultra-low detection levels (3–15 mg/dL). The D-glucose concentration is determined by monitoring the variations of protons level as by-products of the oxidation process when D-glucose (the target) binds with the glucose oxidase bioreceptor [24,78].

Biochemical sensing based-FETs can be carried out either in quasi-real time or in steady-state manners. In the former case, a short pulse of gate voltage is applied to enable high-frequency transient measurements while the drain current is continuously monitored, with the sensing channels being immersed in the measuring solution [18,22,49]. Real-time measurements are beneficial for accessing binding kinetic information. However, measuring binding kinetic data ideally requires a fluidic system to deliver the sample and buffer solutions [45] and is, therefore, more complex to implement. In the steady measurement, the affinity FET sensors are exposed to the sample containing the target molecules to allow for the binding reaction to take place and eventually reach binding equilibrium or saturation state. Drain currents before and after completion of the assay are acquired, and these measurements are used to extrapolate the concentrations of the target analytes based on a calibration curve [14,79].

Typically, the measurement procedure of FET sensors starts with biasing process. To bias metal-oxide-based FET devices, fixed V_DS_ and V_GS_ are applied. There are two main gate-biasing approaches, namely the subthreshold and linear regimes (at or near the transconductance peaks, V_max gm_, Figure 2c) [80,81]. The subthreshold operation of a FET sensor is preferred to improve analytical performance, as in the subthreshold regime, the drain current (I_DS_) response relies exponentially on the gate voltage (V_GS_). Otherwise, I_DS_ varies linearly with V_GS_ in the linear regime. The rationale for operating in the linear regime is based on the argument that a high signal-to-noise ratio can be achieved, thereby providing lower LOD. It is important to note that each operation regime depends on the FET structure, the semiconductor materials, and the measuring conditions. In terms of real-life sensing implementation of a specific nano-FET device, it is essential to determine the most appropriate operating point according to the specific application.

## 3. Development and Integration of Metal-Oxide Nano-FET Biosensor for PoCT

### 3.1. Fabrication of Nanoscale Metal-Oxide Semiconductors: Vapor-Based Approaches

Scaling down metal-oxide semiconductors into the nanoscale increases their analytical performance due to the comparable size of metal-oxide nanostructures to the targeted molecules [82]. Metal-oxide nanostructures, including nanowires (NWs), nanoribbons (NRs), nanobelts, nanorods, and nano-thin films, can be grown using vapor-phase fabrication techniques, which include chemical vapor deposition (CVD) and physical vapor deposition (PVD).

**Figure 3:**
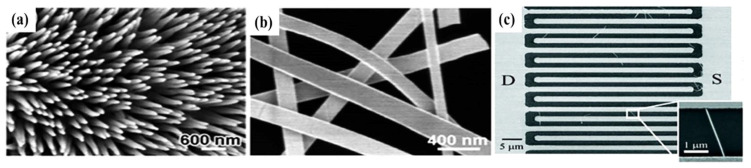
Vapor-phase-grown nanostructures: (**a**) ZnO nanowires; (**b**) SnO_2_ nanobelts; Reproduced from Ref. [83] with permission from The Royal Society of Chemistry. (**c**) An example of randomly distributed In_2_O_3_ NWs between source and drain during device integration. Reprinted with permission from Ref. [94]. Copyright © 2015, American Chemical Society.

Vapor-phase-grown metal-oxide nanostructures. Metal-oxide NWs and nanobelts can be synthesized using vapor processes such as CVD and PVD (Figure 3). As detailed previously, two mechanisms typically govern these fabrication methods: vapor-liquid-solid and vapor-solid mechanisms [83]. CVD synthesizes nanostructures (NWs, nanobelts, or nanorods) through chemical reactions in the vapor phase with the assistance of a noble metal catalyst (e.g., Pt, Au). Different metal-oxide NWs have been synthesized through laser ablation CVD procedure [83,84,85,86]. Here, NWs are grown in a pre-coated substrate (usually Si/SiO_2_ substrate) with a catalyst (either a thin film or nanoparticles). During the reaction of the laser beam with the targeted material, clusters or droplets of the targeted material are generated and form the NW backbone based on the catalyst size in the pre-coated substrate. A drawback of using a thin film catalyst is that this produces significantly different NW diameters, so it lacks reproducibility [15]. On the other hand, monodisperse metal clusters catalysts allow more control over the NW diameters as the nanocluster catalyst’s size guides the formation of the metal-oxide NWs [87,88]. Since the synthesis reaction takes place at a high temperature (>770 °C), NWs grown with CVD are crystalline as reported for SnO_2_ [89,90,91,92], In_2_O_3_ [53,86,93,94], and ZnO [18,44,46]. On the other hand, PVD produces nanostructures by either thermal evaporation or gaseous plasma. In the thermal evaporation process, a high temperature evaporates the material powder under a vacuum. Figure 3a depicts ZnO nanowires with an average diameter of 150 nm fabricated on a silicon substrate by thermal evaporation of a zinc powder at 550 °C. While in the gaseous plasma method, as in sputtering, gaseous ions (plasma) are used to generate vapors of the targeted material under a vacuum by dislodging the atoms or molecules from the solid target. The PVD process normally does not involve using of a catalyst and is governed by vapor-solid mechanisms as in the case of SnO_2_ nanobelts (Figure 3b) [39,95]. Vapor-phase synthesized NWs and nanobelts have crystalline orientation, presenting high electrical performance. However, lack of uniformity, inherent random distribution, and alignment of these metal-oxide FETs during device assembly and integration, as well as limited control over the density of nanomaterials on each sensor channel (illustrated in Figure 3c), are significant shortcomings in view of the development of high-performance FET biosensors for PoCT applications [49,82,96,97]. These technical challenges typically result in low fabrication yields and poor large-scale uniformity that significantly exacerbates the problem of device-to-device signal variations [96,98].

To prepare a functional FET device from metal oxide nanostructures grown in the vapor phase, a patterning/transfer technique is typically required, which aims to transfer the as-synthesized nanostructures to a secondary (receiver) substrate. The patterned semiconductor nanostructures can then be integrated with electrical contacts and isolated using a passivation layer. Shadow masks and conventional photolithography with an etching or lift-off process are mainly used in the patterning step [23,24,99,100,101]. Conversely, dip-coating is the most used method for transferring metal-oxide FETs to the patterned substrate [98]. In this methodology, the as-grown semiconductor materials are transferred from the growth substrate into an organic solvent (usually an alcoholic solution) using ultrasonication. The resulting suspension is then dispensed drop-by-drop onto the secondary substrate. Although there are many reports of successful transfer of CVD/PVD-fabricated metal-oxide nanostructures using dip-coating (e.g., In_2_O_3_ [51,98,102], ZnO [18,44], and SnO_2_ [90,92,103]), this procedure is limited by the substantial damages occurring in the metal-oxide nanostructures during the ultrasonication step. Alternatively, CVD/PVD-fabricated metal-oxide semiconductors can be transferred with direct contact printing [83,92]. In this approach, the nanostructures are transferred by the shear force by directional sliding of the growth substrate over the receiver substrate. The density of the nanostructures on the receiver substrate depends on their thickness on the growth substrate and can be increased by repeating the process.

Vapor-phase thin film deposition techniques. When combined with a patterning process, metal-oxide FET can also be fabricated by top-down vapor-phase thin film deposition approaches. The most commonly used vapor-phase thin film deposition techniques for this purpose include sputtering, atomic layer deposition (ALD), and pulsed laser deposition (PLD).

Sputtering is a widely used thin film deposition technique [23,24,99,100]. Metal-oxide FET fabrication based on sputtering has several merits, namely (1) relatively low-temperature processing (from room temperature to a few hundred degrees), which makes it readily compatible with a wide range of substrates, for example, flexible plastic substrates [104,105], silicon/glass substrate, (2) efficient control of the thickness and morphology of the metal thin films by modulating the sputtering conditions (e.g., sputtering power, time, and gas flow rate), and 3) control of the composition of the deposited film by co-sputtering different material targets [105]. Metal-oxide thin films can be deposited either from the respective metal-oxide targets in an inert atmosphere or from a pure metal target within an oxidative gas environment, which is typically referred to as reactive sputtering [106]. Sputtering of oxide targets in an inert atmosphere is superior due to the simplicity and superior reproducibility of the process, while reactive sputtering is more sensitive to contaminants and process parameters.

**Figure 4:**
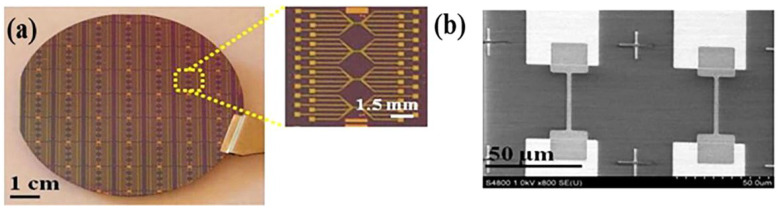
(**a**) Photograph of a 3-inch wafer with top-down fabricated In_2_O_3_ nanoribbon FT biosensors. Inset shows a magnified image of a nanoribbon chip composed of four subgroups of six nanoribbon FETs; (**b**) SEM micrograph of the nanoribbon FETs showing identical device channels precisely positioned on the source and drain areas, reproduced with permission from Ref. [23]. Copyright © 2015, American Chemical Society.

Generally, as-sputtered metal-oxide FET devices using oxide sputtering targets (Figure 4a) have good electrical properties with field effect mobilities >10 cm^2^ V^−1^s^−1^ and an on/off current ratio from 10^4^–10^7^, therefore, they are compatible with high sensing performance [23,24,99]. Sputtering typically produces amorphous thin films of metal oxides, which can be turned into a crystalline structure by a high-temperature annealing process. However, due to the absence of grain boundary in metal-oxides, carrier mobilities are not significantly affected by crystal orientation [23,105].

Atomic layer deposition films are grown by the atomic layer step, thereby providing extremely precise control over the deposited thin film thickness. ZnO NW FETs [103] and ZnO thin film FETs [107] have been successfully prepared using Atomic layer deposition. A limitation of the atomic layer deposition technique is that it is a relatively slow process, and consequently, it is more suitable for the deposition of high-quality metal-oxide dielectric films with well-controlled thickness. For example, Samsung has developed a new 10 nm-class DRMAs with a dielectric layer uniform to a few angstroms using the atomic layer deposition technique [108]. Pulsed laser deposition produces thin films by radiating metal-oxide targets with a high-power pulsed laser beam. Thin films of ZnO (Figure 4b) [109,110], SnO_2_ [111], and In_2_O_3_ [112] have been fabricated using pulsed laser deposition. While pulsed laser deposition yields excellent and high-performance metal-oxide FETs, it is not compatible with scaled-up production due to the low deposition rate, poor film uniformity over a large area, and high capital cost.

### 3.2. Fabrication of Nanoscale Metal-Oxide Semiconductors: Solution-Based Approaches

Most metal-oxide nanostructures/thin films can be synthesized through solution-based routes (e.g., sol-gel [113,114,115], and wet-chemical synthesis [84,114,116]). In this paradigm, the nanoscale metal-oxide elements are subsequently deposited on substrates, for example, using spin coating, spray coating, bar coating, and printing [105,113,117]. When combined with the patterning technique, the solution-based route is cost-effective and more compatible with large-area deposition than vapor-based techniques [113,114,118].

In a typical sol-gel process, a dissolved metal salt precursor is spin-coated or printed directly on a substrate at room temperature. A high-temperature annealing (200–350 °C) step is then used to convert the precursor framework into the desired metal-oxide framework by decomposing and desorbing by-products of the synthesis reaction [86,117,119]. This step determines the metal-oxide FET’s electrical properties. In_2_O_3_ thin films [25,119,120], In_2_O_3_ nanoribbons [38,118], and ZnO thin films [115] have been successfully fabricated by a sol-gel process with electron mobility μ_FE_ > 10 cm^2^ V^−1^ s^−1^, current on/off ratios from 10^4^ to 10^7^, and SS values from 81 mV/decade to 600 mV/decade [25,118,119,120]. High-temperature annealing requirement is a drawback of the sol-gel approach. It restricts the substrate material choice as many polymeric substrates cannot tolerate the required high temperature. Therefore, new strategies to lower the annealing temperature are needed to combine this approach with flexible substrates, for example, using novel precursors and/or developing innovative annealing methods [115,117].

**Figure 5:**
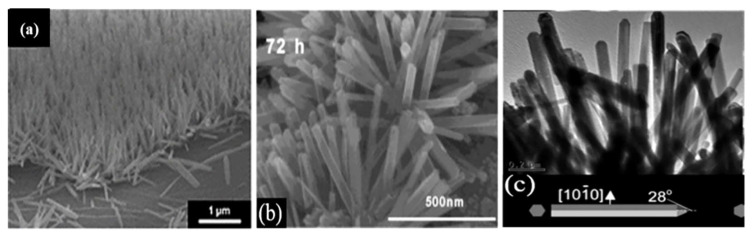
Examples of solution-synthesized nanostructures: (**a**) 600 tilted cross-sectional FE-SEM images of vertical ZnO NWs grown on a reduced graphene/PDMS substrate. Reprinted with permission from the Royal Society of Chemistry (Ref. [116]); (**b**) HR-TEM image of SnO_2_ nanorods. Reprinted with permission from Ref. [123]; (**c**) TEM images of ZnO nanorods. Reprinted with permission from Ref. [122].

The wet chemical synthesis route is another solution-based technique to synthesize crystalline metal-oxide nanostructures compatible with the preparation of FET sensors. In wet chemical growth processes, metal-oxide seeds are first deposited either by spin-coating the seed solution or by sputtering a metal-oxide layer on a substrate [47,121]. The modified substrate is then exposed to a salt solution of the metal at an elevated temperature (~200 °C) to synthesize the nanostructures. This is a cost-effective and up-scalable way to synthesize crystalline metal-oxide nanostructures. For example, vertically aligned ZnO nanorods [46,84,122], ZnO NWs [116], and SnO_2_ nanorods [123] have been successfully fabricated using this approach (Figure 5).

### 3.3. Surface Functionalization of Metal-Oxide-Based FET Sensors

A range of biorecognition approaches, including enzyme/substrate, antibody/antigen interactions, and nucleic acid hybridization, has been exploited to impart selectivity to biosensors for a specific molecular target [124]. These bio-affinity recognition methods have been successfully implemented to realize experimental FET biosensors for testing many diseases with high prevalence, such as cancers (e.g., protein biomarkers) [21,113], cardiovascular diseases (e.g., protein biomarkers) [125,126], infectious diseases (e.g., nucleotide biomarkers) [37], and diabetes (e.g., protein and enzyme biomarkers) [24].

**Figure 6:**
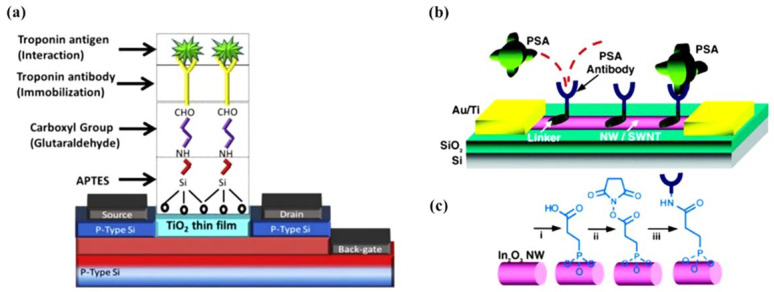
(**a**) A TiO2 thin film-based biosensor’s surface functionalization process involves silanization with APTES, reaction with bifunctional linker (glutaraldehyde), and covalent antibody conjugation. Reprinted from Ref. [144] with permission from Elsevier. Copyright © 2017, Elsevier BV All rights reserved. (**b**) Schematic diagram of FET-based In_2_O_3_ NW biosensors for the prostate-specific antigen (PSA). Monoclonal antibodies (Abs) are anchored to the surface of the NWs and serve as specific recognition groups for PSA. (**c**) Reaction sequence for the functionalization of In_2_O_3_ NW:  i, 3-phosphonopropionic acid deposition; ii, DCC and N-hydroxysuccinimide activation; iii, PSA-Abs incubation. Reprinted from Ref. [142] with permission from ACS. Copyright © 2005, American Chemical Society.

An essential step in preparing a nano-FET biosensing device is introducing a molecular bioreceptor with high and specific binding affinities to the target of interest on the surface of the FET. Metal-oxide-based FETs are, therefore, typically functionalized first with a chemical agent to enable covalent immobilization of the specific bioreceptors on their surface. In this regard, organosilanes and phosphonic acids are widely used. Organosilanes covalently bind to many metal-oxides such as In_2_O_3_ [38,78,100,127], SnO_2_ [39], ZnO [44], SiO_2_ [128,129], Fe_3_O_4_ [130], and β-Ga_2_O_3_ [131]. Presently, 3-aminopropyltriethoxysilane (APTES), 3-mercaptopropyltrimethoxysilane, and 3-(trimethoxysilyl) propyl aldehyde are the most used amongst the organosilanes. Silanization is carried out either in vapor or liquid phases (Figure 6a) using a mixture of ethanol/water 95%/5% (*v/v*), or toluene [128,132,133]. Prior to surface functionalization, metal-oxide surfaces are often activated using UV-ozone or oxygen plasma. This activation step generates –OH groups on the surface, facilitating the reaction with the organosilanes. The mechanisms and characteristics of metal-oxide functionalization with organosilanes have been reported in detail elsewhere [128,130,134,135]. In the case of APTES, the amine functional groups can be subsequently reacted with a mono or hetero bifunctional linker, such as glutaraldehyde, to introduce reactive groups able to covalently conjugate bioreceptors including DNA probes [136], antibodies [21,137], proteins [137], or enzymes [18]. After immobilization, unreacted groups (e.g., CHO) are usually blocked, and the surface is passivated toward reducing non-specific adsorption events [132,138,139].

Phosphonic acids are also known to bind to the surface of many metal-oxides covalently [75,140,141]. For example, In_2_O_3_ NWs have been functionalized using 3-Phosphonopropionic acid with terminal carboxylic linker groups, which could subsequently be activated using carbodiimide chemistry to covalently immobilize antibodies and primary amine-terminated DNA probes, as shown in Figure 6 [142,143]. Phosphonic-acid-based approaches are less sensitive to moisture than organosilanes (facilitating storage and practical applications) and less prone to self-condensation. In addition, phosphonic-acid-based monolayers are more resistant to hydrolysis than organosilanes [140].

## 4. Tailoring Metal-Oxide Nano-FETs toward Point-of-Care Testing Applications

PoCT as a diagnostic device must be fast time-to-result (ideally in less than an hour), cost-effective, portable, instrument- and technician-free, robust enough in the implementation environments (i.e., weather and shelf-time), and sensitive. Recent advances in metal-oxide nano-FET make it possible to meet the PoCT requirements satisfactorily.

The first application of metal-oxide nano-FET for PoCT was reported by Chang et al. [98]. They developed a nano-sensor system based on indium oxide NWs and demonstrated an early prototype of a finger-prick device for the PoCT of cancer biomarkers. This pioneering concept of sample-in-signal-out was successfully applied to detect two epithelial ovarian protein cancer biomarkers, namely cancer antigen 125 (CA-125) and insulin-like growth factor II (IGF-II), in whole blood obtained from a finger prick. The FET sensor was connected to a microfilter to filter blood cells and generate plasma. LODs of 10 U/mL in serum were achieved for both cancer biomarkers, which is well below the clinically relevant concentrations (100–275 U/mL). Following this pioneering study, they investigated another In_2_O_3_ NR FET biosensing platform to detect the human immunodeficiency virus type 1 (HIV-1) p24 proteins for enabling PoC early diagnosis of HIV [23]. The LOD was 20 fg/mL (250 viruses/mL), which is three orders of magnitude lower than a commercial ELISA. The same platform was also successfully applied to detect three protein biomarkers for myocardial infarction diagnosis, including troponin I (cTnI), creatine kinase MB (CK-MB), and B-type natriuretic peptide (BNP) with LODs well below clinically relevant cut-off concentrations (1 pg/mL for cTnI, 0.1 ng/mL for CK-MB, and 10 pg/mL for BNP) [99]. Sample collection to result-time was only 45 min, illustrating the potential of this platform for PoCT of acute myocardial infarction.

ZnO nanorod-based FET biosensors have also shown significant potential for PoCT applications [45,47]. For example, measurement of PSA/1-antichymotrypsin with a LOD of 1 fM in just 1 min was achieved using a 3D bio-FET comprising vertically aligned ZnO nanorods [45]. Multiplexed detection of glucose, cholesterol, and urea in mice and dog blood was also achieved with ZnO nanorod FETs [47]. A SnO_2_ nanobelt FET biosensor was also reported for troponin-I detection as a biomarker for myocardial infarction with a LOD of 100 pM (~2 ng/mL) [39].

### 4.1. Sample Processing Integration

Besides a few notable exceptions, FET biosensors remain to date mostly in the research and development realm, with R&D focussed mainly on sensor development [145,146,147] and bioassay/detection elements [144,148,149]. However, important underlying issues associated with sample processing typically required prior to/during PoC testing have received far less attention, contributing to the limited real-life deployment of FET-based PoC platforms.

**Figure 7:**
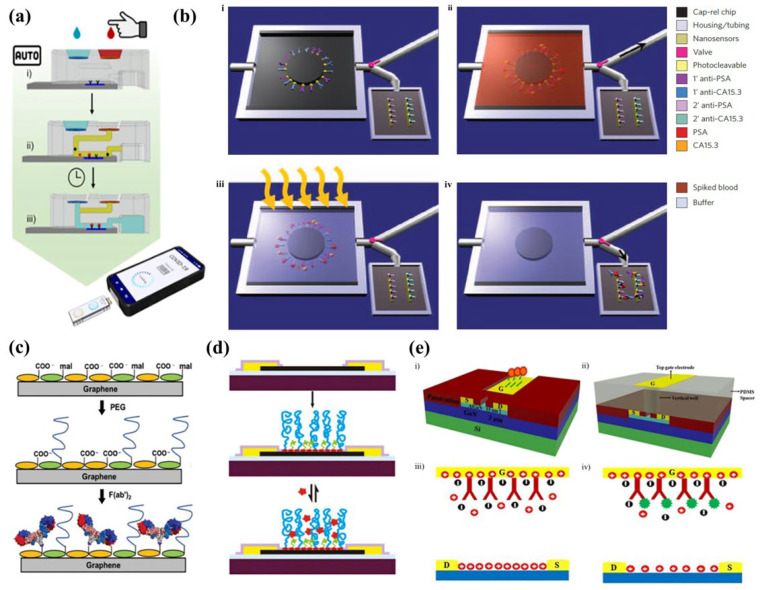
(**a**) Schematic of the 3D-printed hydrostatic pressure-driven PoC sample processing platform performing filtration and washing on chip. American Chemical Society Copyright © 2022 [37]. (**b**) Schematic of SiNWs microfluidic purification chip performing protein purification, then photocleaving the crosslinker to release the purified protein into the sensing area. Adopted from Ref. [151] (**c**) Schematic showing Pyrene-modified graphene functionalized with thiol–PEG, then with the F(ab′)2 antibody fragment against the thyroid-stimulating hormone. Reprinted from Ref. [168] with permission from Advanced Materials Technologies Copyright © 2018. (**d**) Schematic of a graphene FET device with PEG and a small-molecule spacer for non-specific and specific detection of the analyte directly in physiological samples [167]. (**e**) Schematic model of AlGaN/GaN HEMT with the gate electrode functionalized with respective antibody/aptamer and measurement of impedance to allow novel sensing method in the physiological sample directly. Reprinted from Ref. [76] with permission from Springer Nature Copyright © 2017.

The need for sample processing in FET biosensors lies within the complexity of biofluids typically used for PoCT, including blood, saliva, urine, and to a lesser extent sweat. Such biofluids vary substantially in their composition (e.g., pH, protein concentration, ionic strength) and typically interfere with biological assays and/or analytical performance of the sensors. Various biofluid processing approaches are commonly used with FET biosensors, including blood filtration/desalting [79,132,139,150], centrifugation/washing [22], chemical pre-treatment, microfluidic biomarker pre-enrichment [151], and novel sensing methodology [152]. These approaches typically rely on the intervention of trained staff and the use of external analytical equipment (e.g., centrifuge, micropipette, pump), which is often challenging in PoC settings. In terms of simplifying PoCT and eliminating analytical errors typically associated with sample manipulation by operators, integrated approaches have been actively explored. This includes operation by benchtop instrument through supply energy or timed triggering mechanism [153,154,155,156], internalized vacuum/chemical reactions, and capillary pump [157,158,159,160]. Various strategies have been investigated to integrate sample processing features within FET biosensing platforms for enabling PoCT. For example, Cheah et al. developed a hydrostatic pressure-driven 3D-printed platform incorporated with a high-yield blood-to-plasma separation module and a delay valve designed to terminate the assay at a specific time (Figure 7a) [37]. This approach was demonstrated for a SARS-CoV-2 nucleocapsid antigen immunoassay with indium oxide NRs FET sensors, allowing intervention-free sample processing and assay termination in a single device. The SiNWs microfluidic purification chip developed by Stern et al. relies on a two-stage approach with distinct components within the sensor to perform the purification of whole blood and detection on a single sensor [151]. This chip captures the target biomarkers from blood samples, removes unwanted blood components, then releases the biomarkers into the purified buffer for measurement, as illustrated in Figure 7b.

The sample ionic strength is especially significant in the context of the FET platform as the Debye screening length (λ_D_) is a key parameter dictating the sensitivity to a given analyte [37,161,162,163,164]. Briefly, the presence of counter ions in the measurement solution effectively screens the charge of the analyte that can be sensed at the FET surface, with the screening length in physiological solutions being below 1 nm [165,166]. To circumvent this issue, molecular probes with smaller dimensions have been used, including cleaved antibodies and aptamers. Alternatively, polymeric biointerfaces have been shown to extend the λ_D_ of the sensor and consequently enable FET measurement at high ionic strength. For example, Gao et al. utilized polyethylene glycol (PEG), small-molecule spacer, and aptamer on Graphene FET biosensors (Figure 7d) to directly measure prostate-specific antigen (PSA) in physiological conditions [167]. Andoy et al. also demonstrated the use of PEG coatings to enable direct measurement of thyroid-stimulating hormones in whole serum using Graphene FET biosensors (Figure 7c) [168]. Chu et al. utilized AlGaN/GaN high electron mobility transistors and a novel sensing methodology through the measurement of impedance and capacitance to directly measure in high ionic strength physiological samples without sample processing [76]. Unlike conventional FET sensor operation, the molecular probes are functionalized on the gate electrode (Figure 7e) rather than on the FET itself, and the target is measured through the voltage drop between the impedance and dielectric layer to determine the capacitance of the solutions. This approach is promising for PoCT due to its short turnaround time and ultra-high analytical performance, but it remains to be validated.

### 4.2. Analytical Validation and Regulatory Approval of Point-of-Care Testing Devices

**Figure 8:**
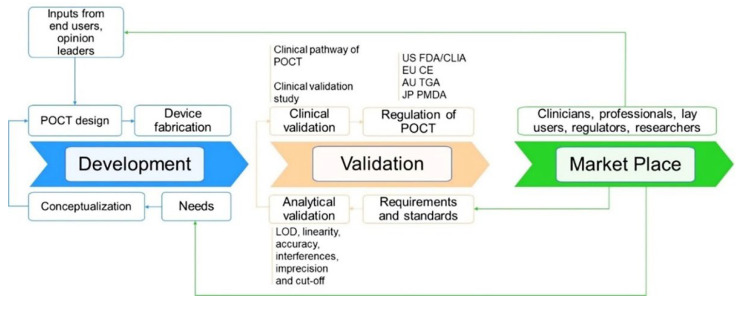
Development, validation, and regulatory pathway of a biosensor PoCT technology and their interconnection. Abbreviation: US FDA—United States Food and Drug Administration, EU CE—European Conformite Europeenne, AU TGA—Australia Therapeutic Goods Administration, JP PMDA—Japan Pharmaceuticals and Medical Devices Agency.

As with all medical devices, PoCT devices should be thoroughly validated and subjected to regulatory approval prior to being implemented and commercialized (Figure 8). A critical aspect here is to demonstrate that the device’s real-world performance is acceptable and that it complies with regulatory standards and requirements of a given jurisdiction (e.g., United States-United States Food and Drug Administration, FDA; European Union-European Conformite, CE; Australia-Australia Therapeutic Goods Administration, TGA; China-National Medical Products Administration (NMPA); Japan-Japan Pharmaceuticals and Medical Devices Agency, PMDA). The bench-to-bedside journey for PoCT devices is complex, expensive, and undoubtedly associated with a “valley of death”. Cross-disciplinary collaboration with all key stakeholders is, in most cases, essential to successfully navigating the various regulatory processes required for developing and commercializing PoCT devices.

Validation of the analytical performance of PoCT platforms. Validating the analytical performance is a critical step in the development of any IVD medical device, including PoCT biosensors. A consideration specifically relevant to the PoCT platform is that it must be designed to achieve the required level of performance, taking into account the skills and the means available to the intended users, for example, lay persons. Testing should therefore consider the variation that can reasonably be expected in the user’s technique and environment. The assessment of the analytical performance should include all aspects relevant to the intended use of the PoCT device, including analytical sensitivity (e.g., the limit of detection or limit of quantitation, inclusivity) and specificity (e.g., interference, cross-reactivity), accuracy (derived from trueness and inter/intra assay precision) and linearity (as applicable) [52,169,170].

In the case where an approved reference method is available, the difference between the PoCT device and the comparative predicate method should be determined to establish the trueness of the PoCT device. In this case, samples covering the clinically meaningful range should be used and measured as defined in relevant standards such as the Clinical Laboratory Standards Institute EP09-A3 guidelines.

Establishing the clinical performance and utility of PoCT biosensors. Clinical validation is a process that evaluates the performance of a PoCT device to deliver data that is correlated with a particular clinical condition/physiological state for the target population and intended user. A key aspect of the regulatory process is, therefore, to demonstrate that the PoCT device effectively informs on a patient’s current or future state or evaluates changes in the patient’s state. Assessing the clinical performance and utility of the test is also central to its successful implementation and to defining the associated reimbursement model. In practice, clinical validation is usually performed by sponsors of new medical devices or health professionals [171].

Regulation of PoCT biosensor. For most PoCT diagnostic devices, regulatory approval is mandatory prior to the product launching to the market. In addition, post-market surveillance is also necessary and provides additional insight into the patient population and the use of the device in the real world. The regulatory oversight is not only applied to the PoCT device itself but also to any associated software, consumables (regents, calibrator), and user instructions [169].

Medical PoCT biosensors are classified under low and medium risk to the user, and low complexity tests are usually streamlined in most of the regulatory landscapes, while higher risk tests are subjected to more stringent regulation. For instance, in the US, most class I and II medical devices under the Clinical Laboratory Improvement Amendments category are either waived from the FDA pre-market approval process or can obtain a pre-market notification clearance, aka 501[k] clearance [172,173]. For class III devices with significantly greater risks to patients, a full pre-market approval needs to be fulfilled with intensive clinical trial evidence.

It is worth noting that new PoCT biosensors where there are no approved/predicated technologies are automatically classified as Class III and must undergo a full pre-market approval process in the US FDA landscape. On the other hand, class III PoCT biosensors with existing predicated technologies can apply to be accessed as class II devices [173]. Similarly, many low-to medium-risk PoCT devices in Europe can be CE mark self-certified by providing appropriate technical documentation of conformity; only high-risk devices require the involvement of notified bodies. In general, it is estimated that completing a full clinical validation program toward regulatory approval of a new PoCT biosensor takes around 2 to 3 years and costs several million [174]. Due to the high cost and the complexity of the regulatory landscape, most new medical PoCT biosensors enter the market from venture-backed companies and large corporations rather than academic organizations.

## 5. PoCT Adoption Barriers and Limitations

The relatively poor adoption of PoCT can be explained by performance and economic issues compared to more conventional testing in centralized laboratory facilities. In addition, cultural and organizational issues are also commonly identified as barriers to implementation [175]. Finally, while metal-oxide FET sensors inherently address some of the key challenges faced in designing and implementing PoCT technologies, specific challenges remain to be solved.

### 5.1. PoCT Performance Issues

The limited performance—real or perceived—of some PoC tests (i.e., specificity, sensitivity, and precision) compared to tests performed within centralized laboratory facilities may impede their adoption and utility. Poor PoCT device performance, particularly “false negative results”, can have serious consequences. Performance issues are also inherently linked to the issues associated with the quality control of PoCT devices. For example, six of eight PoC tests for Hemoglobin A1c yielded clinically unacceptable reproducibility, although certified by the National Glycohemoglobin Standardization Program (NGSP) [176]. The performance issue of PoCT has been vividly illustrated recently during the COVID-19 pandemic. PoCT undoubtedly played an essential role in the rapid tracking of the SARS-CoV-2 virus and as such greatly contributed to the containment of the pandemic [177]. However, the performance of COVID-19 rapid antigen PoCT devices has not matched that of tests conducted in centralized laboratories (Table 2). For instance, compared with real-time RT-PCR, the Wondfo^®^ test shows high specificity (95%) with noticeably low performance (63%) using serum samples [178]. The CLINITEST^®^ test presents good performance (80%) with almost the same specificity as Wondfo^®^ devices (97%) for symptomatic patients only when testing within five days of symptoms onset [179]. Interestingly, the SiennaTM device shows high analytical performance (90%) with a 100% specificity for symptomatic patients only, and the symptom’s onset date was unknown [180].

### 5.2. Challenges and Considerations for Metal-Oxide FET-Based PoCT

As noted above, metal-oxide FETs are cost-effective devices derived from the availability of well-established fabrication protocols compatible with scaled-up manufacture. In addition, their performance is high enough to meet the requirements of most PoCT applications. They, therefore, provide an excellent compromise between ultra-high-performance solid-state sensors that typically suffer from limited manufacturability and low-sensitivity sensors that can be readily mass-produced.

Despite these advantages, several issues should be considered. A critical challenge associated with the implementation of metal-oxide FET sensors is batch-to-batch performance uniformity. To mitigate this issue, it is essential to consider all factors that affect their performance. For example, it has become evident that the characteristics of metal-oxide semiconducting materials are controlled not only by their structure and geometry but also by the presence of functional defects and their crystal structure. Most importantly, oxygen vacancies play a crucial role in determining the physical properties of metal-oxide FET sensors [181] and should be carefully controlled throughout the manufacturing process. However, the preparation of high-performance metal-oxide FETs might increase their cost beyond what is economically acceptable. This may be addressed by implementing an extended gate configuration, which reduces cost by increasing the FET sensor life-time and also its stability. On the other hand, extended gate geometry and design should be considered carefully to avoid detrimental effects on the sensor performance.

Importantly, FET-based PoCT devices should have a small footprint to enable integration with signal processing systems. The inherently small size of FET sensors increases compatibility with wearable technologies, which is currently attracting a huge amount of research. In addition, impedance matching between the subsystem units should be considered, as it affects the signal capture within these units’ signal-to-noise ratio level [124]. One should also consider the need to integrate a reference electrode within the FET sensors, which presents a significant challenge. The issue of reference electrode miniaturization and integration has been reviewed in detail elsewhere and we refer the reader to the relevant literature [182].

Finally, an often-overlooked consideration is the issue of the packaging and shelf-life of FET-based sensors. The environmental stability of the metal-oxide FET itself and that of the biointerfacial layers containing the bioreceptors should be considered and optimized to ensure compatibility with PoC settings, where substantial variations in storage conditions are likely to occur.

### 5.3. Economic Considerations

The economic dimension is a prominent barrier to the adoption of PoCT. In general, the cost per test is higher for PoCT than that associated with conventional batch testing in centralized laboratories. For example, it has been reported that the cost of glucose and blood gas/electrolyte testing is 1.1 to 4.6 times higher for PoCT compared to testing in a centralized laboratory [183]. Along the same line, 80% of clinical staff respondents agreed (or strongly agreed) that the cost of PoCT for cardiac markers impedes their adoption [184].

Regarding the PoCT implementation cost, there are both direct and indirect costs associated with the implementation of PoCT. While direct costs are relatively straightforward to evaluate [185], indirect costs related to operational aspects, such as staff training, quality assurance, laboratory accreditation, etc., are often difficult to cost. But while accurate data is lacking, the implementation cost of PoCT is likely higher compared with testing conducted within centralized facilities. For example, the specific IT cost associated with the implementation of cardiac marker PoCT devices was estimated to be around £20,000 for a clinical center [186].

Finally, it is worth noting that, in most cases, reimbursement schemes are not adjusted to cover the direct and indirect additional costs associated with PoCT [187]. For instance, the UK’s central fund for clinical pathology provides the same fee for PoCT as for centralized testing, regardless of the workload or the patient care pathway [188]. This is likely a further barrier to adoption, and reimbursement should consider the patient care pathway to overcoming it.

## 6. Conclusions and Future Perspectives

This review has discussed the most common metal-oxide FETs fabrication routes, material selection and considerations, surface functionalization, and emerging biosensing applications in the PoCT area. Daunting challenges remain, however, to be addressed so that metal-oxide FET technology can be reliably translated into real-life applications.

The first challenge is the current significant device-to-device signal variability, which exists even for devices originating from the same fabrication batch. These variations could be significantly reduced by using nanoribbons and thin-film FETs instead of nanowires. These architectures allow much better control over the lateral dimensions and do not affect the device’s performance. Secondly, implementing metal-oxide FETs in the PoCT application area requires careful choice of fabrication procedure, sample collection/processing integration approaches, and readout methods. Therefore, striking the right balance between the privileges and shortcomings of each process and minimizing user involvement.

Despite challenges, significant progress has been made in metal-oxide FET-based biosensors recently. With their ultralow LODs, exquisite sensitivities, quantitative, and label-free sensing, metal-oxide FET promises to play a significant role in the bio-diagnostic field.

## Figures and Tables

**Table 1:** Commonly used metal-oxide semiconductor materials-based FET for bio/chemical sensing applications.

Oxide Semiconductor	Band Energy (eV)	Reported Sensing Applications	Range of the Target Detection	Ref.
Zinc Oxide (ZnO)	3.4	Biomarkers (prostate cancer antigen, streptavidin, uric acid, glucose, urea, cholesterol, riboflavin).	fM-M	[18,44,45,46,47,48]
Tin oxide (SnO_2_)	3.6	Cardiac troponin I, biotinylated protein, pH, and NO_2_ gas.	3–10 (pH) <15–100 (gas)	[49,50]
Indium oxide (In_2_O_3_)	3.5–3.6 (direct) 2.5 (indirect)	Biomarkers (DNA, prostate cancer antigen, glucose, dopamine, p24 protein, PlGF protein, Cardiac troponin I (cTnI), creatine kinase MB, and B-type natriuretic peptide, cholesterol), pH and gases (NO_2_, NH_3_).	4–9 (pH) fg-g	[14,23,24,25,40,51,52,53,54,55]
Indium tin oxide (ITO)	3.2–4.2	DNA and pH.	2–12 (pH) fM-μM	[56,57,58]
Gallium oxide (Ga_2_O_3_)	4.8	pH, Ultraviolet photodetector, and power transistor application (energy-efficient power switches).	1–11 (pH)	[59]
Copper oxide (Cu_2_O)	2.17	Photodetection.	-	[60,61]
Hematite (α-Fe_2_O_3_)	2.1	Glucose.	μM-mM	[62,63]
Cerium oxide (CeO_2_)	3.2	pH.	2–12 (pH)	[64,65]
Vanadium pentoxide (V_2_O_5_)	2.3	pH.	2–12 (pH)	[66,67,68]
Manganese Oxide (MnO_2_)	2.2	Ascorbic acid and lactate.	In mM range	[69,70]
Cobalt oxide (Co_3_O_4_)	1.5–2.2	Cardiac troponin T (cTnT).	In μg range	[71]
Titanium dioxide (TiO_2_)	~3.2	Ultraviolet detection and pH.	1–13 (pH)	[72,73]

**Table 2:** Comparison of some approved COVID-19 rapid antigen tests.

PoCT	Approval	Sample	Sensitivity (%)	Specificity (%)	Price ($)	Ref.
Wondfo^®^	TGA ^a^	Serum	63	95	4.1	[178]
CLINITEST^®^	EUA ^b^	Nasal mucus	80	97	3.76	[179]
SiennaTM	EUA ^b^	Nasal mucus	90	100	4.99	[180]

^a^ Australian Therapeutic Goods Administration (TGA) has approved the test for inclusion in the Australian Register of Therapeutic Goods (ARTG). ^b^ In the USA, this product has not been FDA cleared or approved but has been authorized under an Emergency Use Authorization (EUA).

## Data Availability

Not applicable.
